# Beyond risk factors: a mixed-methods study of interpersonal dynamics in suicide attempts and reattempts from police narratives

**DOI:** 10.1186/s40352-026-00443-y

**Published:** 2026-08-06

**Authors:** Sarah Al Falatah, Jennifer Tostlebe, Sadaf Hashimi

**Affiliations:** School of Criminology and Criminal Justice, University of Nebraska at Omaha, Omaha, USA

**Keywords:** Mental health, Police data, Repeated suicide attempts, Risk factors, Qualitative thematic analysis

## Abstract

**Background:**

Each year, approximately 1.5 million Americans attempt suicide, and repeated attempts pose heightened risks for individuals, families, and communities. These attempts can result in long-lasting injuries, isolation, and psychological distress. Research relies heavily on structured quantitative data, limiting insight into interpersonal and situational dynamics. Although mental health concerns are well-established risk factors for suicidal behavior, it remains unclear which concerns predict reattempts. We address these gaps using four years (2018–2021) of police service-call data (*N* = 2,659 incidents) from one Midwestern city. Using a mixed-methods design, we conduct thematic analysis of officer narratives to explore the situational context of suicide attempts and estimate logistic regression models to examine whether mental health concerns are associated with repeated attempts.

**Results:**

Qualitatively, seven themes, including relationship conflict, health issues, and general life stressors, are identified, reflecting interpersonal dynamics consistent with the Interpersonal Theory of Suicide. Quantitatively, however, police-documented mental health concerns are not significantly associated with repeated attempts. These null findings should be interpreted cautiously, as they may reflect limitations in police documentation.

**Conclusions:**

Together, these findings highlight the value of mixed-methods approaches for police data research, the need for standardized crisis documentation practices, and the importance of improving officer training and integrating mental health professionals into crisis response. They also underscore the importance of understanding the interpersonal context to aid suicide prevention and intervention efforts.

## Background

In 2023, over 49,000 people in the United States died by suicide—an age-adjusted rate of 14.1 per 100,000 (Centers for Disease Control and Prevention [CDC], 2025). Suicide attempts are far more common, with roughly 1.5 million Americans (600 per 100,000) engaging in intentional but ultimately non-fatal efforts to end their own lives (CDC, 2024a). Among these, repeated attempts represent a particularly acute concern: individuals who survive a first attempt face a higher risk of attempting again and eventually dying by suicide (e.g., de la Torre-Luque et al., 2023; Ribeiro et al., 2016). Initial attempts and reattempts also heighten psychological and physical harms for individuals, and affect families (e.g., trauma, guilt, isolation), workplaces, communities, and first responders (Bommersbach et al., 2023; Hvidkjær et al., 2021; Wayland et al., 2021; Weissinger et al., 2023).

Although empirical research has identified broad risk factors for suicidal behavior such as psychiatric disorders, self-injury, and prior suicide attempts (e.g., Franklin et al., 2017; Kheibari et al., 2023), questions remain about which specific mental health concerns are associated with repeat attempts. Prior work consistently links depression, bipolar, anxiety, and psychotic disorders to reattempts, though effect sizes tend to be moderate and vary across samples and methodologies (Abascal-Peiró et al., 2023; de la Torre-Luque et al., 2023). However, most studies have relied on structured clinical assessments or self-report measures, which, while rigorous, tend to focus on psychiatric variables alone and overlook the situational context in which attempts occur. Specifically, this focus risks obscuring acute stressors and interpersonal dynamics that shape *whether* and *why* individuals reattempt suicide.

The Interpersonal Theory of Suicide (IPTS; Joiner, 2005; Van Orden et al., 2010) offers a useful lens for understanding these dynamics by highlighting how thwarted belongingness and perceived burdensomeness can intensify suicidal crises, particularly for individuals with repeated attempts who may have acquired greater capability for suicide. Still, this framework is difficult to operationalize from psychiatric variables alone. Qualitative accounts, especially those captured at or near the time of crisis, can fill this gap by revealing how individuals interpret their circumstances, reasons for their distress, and what interpersonal factors were present in the moment of a crisis (Biddle et al., 2013; Hjelmeland & Knizek, 2010; Smith & Osborn, 2015). However, such narratives are rarely incorporated into large-scale analyses, leaving an important gap between what quantitative analyses can detect and what is happening near the moment of a suicide attempt.

We draw on a dataset of 2,659 suicide attempt incident reports involving 2,240 individuals recorded by one Midwestern police department between 2018 and 2021 to examine how mental health concerns and interpersonal dynamics shape suicide attempts. Each report contains two components: an officer narrative describing the circumstances of the attempt and an administrative section capturing standardized demographic and incident information. Because these data are generated by responding officers rather than mental health professionals or individuals experiencing the crisis themselves, they provide insight into how suicidal behaviors come to the attention of law enforcement and are documented and interpreted within law enforcement contexts, while also offering a unique perspective on the interpersonal and situational dynamics observed during police encounters. 

We employ a mixed-methods approach that centers the thematic analysis of officer narratives, complemented by logistic regression models using administrative data. Qualitatively, we examine how suicide attempts are described and contextualized in officer narratives, including situational factors at the scene and statements from individuals, friends, and family members when documented. These narrative data provide insight into the interpersonal and situational dynamics of suicidal crises that are often absent from structured datasets. Quantitatively, we examine whether police-documented mental health concerns are associated with repeated suicide attempts. Using the IPTS as an interpretive framework, we situate these quantitative findings within the interpersonal and situational dynamics captured in the narratives, offering a more complete account of the contexts that shape suicidal crises.

### Theoretical frameworks for understanding suicide attempts

To understand why individuals attempt suicide, and more specifically why some repeatedly attempt, it is useful to consider the theoretical frameworks that guide empirical research on suicide. Scholars have long sought to explain suicidal behavior across sociological and psychological perspectives. From a sociological perspective, Durkheim’s (1952) theory of anomie argues that sudden disruptions in society weaken the shared norms that help regulate behavior. As a result, individuals may feel disconnected and purposeless, increasing their vulnerability to self-harm as they attempt to satisfy desires that no longer have clear social limits. This framework implies that strengthening social bonds and community cohesion are central to suicide prevention. From a psychological perspective, Shneidman (1993) emphasized *psychache*, which is an unbearable psychological pain that drives suicidal desire. Suicide, in this view, represents an attempt to reduce overwhelming suffering. As such, Shneidman advocated for prevention strategies that address each person’s subjective emotional experience. Together, these frameworks highlight that social disconnection and psychological suffering are central to suicidal crises. Therefore, understanding why people attempt suicide requires paying close attention to social and interpersonal context.

Building on and extending these ideas, Joiner’s (2005) IPTS offers an integrative framework that bridges sociological and psychological accounts of suicidal behavior. The theory centers on two interpersonal psychological states (Van Orden et al., 2010): *thwarted belongingness*, which captures the painful experience of social alienation, or an absence of meaningful connection even in the presence of others, and *perceived burdensomeness*, which reflects the belief that one’s existence imposes costs on their loved ones, caregivers, or friends, and that these individuals would be better off without them. The IPTS proposes that when both states are experienced simultaneously and feel permanent, suicidal ideation intensifies. However, challenging life events alone do not inevitably lead to suicidal ideation. Rather, it is how individuals interpret those experiences, particularly in the presence of mental health concerns, that may intensify feelings of social disconnection and burdensomeness. For example, a person struggling with depression who loses their job may come to believe that no one truly understands what they are experiencing and that they have become a burden to their family. These interpretations may reinforce feelings of thwarted belongingness and perceived burdensomeness, even when social support is available.

However, ideation alone is insufficient to produce suicidal behavior; the IPTS states that an *acquired capability for suicide* must also be present. Capability for suicide refers to a reduced fear of death and elevated tolerance for physical and psychological pain (Joiner, 2005; Van Orden et al., 2010). Acquired capability develops through repeated exposure to painful experiences that diminish fear of death and increase tolerance of pain. Critically, the IPTS places attention on who is at risk, as well as the interpersonal and situational conditions that generate suicidal crises. Therefore, while interpersonal and situational dynamics shape the context of suicidal crises, individual-level factors, including an acquired capability, remain central to understanding who is most vulnerable in the first place.

### Mental health concerns and suicide reattempts

The CDC (2024b) categorizes suicide risk factors into four domains: individual, relationship, community, and societal. Among these, individual-level factors such as demographic characteristics, prior behaviors, and mental health conditions, are often the most direct predictors of suicidal behavior. These patterns underscore the importance of identifying characteristics that distinguish those who (re)attempt suicide from those who do not. Research consistently shows that females (e.g., Bommersbach et al., 2022), younger individuals (e.g., Nock et al., 2008), and Alaska Native/American Indian and White individuals (e.g., Carter et al., 2022) tend to have higher rates of suicide attempts, including repeated attempts (Abascal-Peiró et al., 2023; Oquendo et al., 2024). Although demographic characteristics describe broad group patterns and may aid in risk stratification, they offer limited guidance for intervention.

Another individual risk factor, mental health concerns, is particularly important because they are both strongly predictive and amenable to intervention, making them a central target for prevention efforts (Nock et al., 2010). Current psychiatric symptoms and a history of mental health concerns, including mood, and anxiety disorders, are associated with both first and repeat suicide attempts, even when accounting for demographics and other risk factors (Abascal-Peiró et al., 2023; de la Torre-Luque et al., 2023; Liu et al., 2017). Suicide attempts also frequently co-occur with, or are preceded by, a range of emotional and psychological struggles (e.g., worry, sadness, anxiety, guilt, and depression) that can improve with treatment (Hvidkjær et al., 2021; Wagner et al., 2021). Given that approximately one in five people in the U.S. experience a mental health condition each year (SAMHSA, 2022), and that 70–80% of individuals who attempt suicide have a documented history of mental illness (e.g., Franklin et al., 2017; Nock et al., 2010), these conditions create a high-risk group.

Still, despite the well-established connection between mental health and suicide attempts, it remains unclear which specific mental health concerns are most strongly related to repeated attempts. In particular, anxiety disorders and mood disorders (e.g., depression and bipolar disorder) are consistently linked to reattempts and are among the most common and clinically important conditions in the general population (Abascal-Peiró et al., 2023; de la Torre-Luque et al., 2023; Kessler et al., 2005). Two recent meta-analyses reinforce this idea. Abascal-Peiró et al. (2023) synthesized 69 studies (*N* = 94,889) and found small-to-moderate associations between reattempts and bipolar disorder (standardized mean difference [SMD] = 0.48, *p*<0.001), depression (SMD = 0.33, *p*<0.001), and anxiety disorders (SMD = 0.29, *p*<0.001). De la Torre-Luque and colleagues (2023) reported similar patterns across 110 studies (*N* = 248,829), with mood disorders (e.g., depression and bipolar disorder; odds ratio [OR] = 1.57, *p* < 0.05), and anxiety disorders (OR = 2.62, *p* < 0.05) both associated with repeat attempts. Notably, the relative strength of these associations differs across studies, with bipolar disorder emerging as the strongest predictor in one analysis (i.e., Abascal-Peiró et al.) and anxiety disorders showing the largest association in the other (i.e., de la Torre-Luque et al.).

Clinically, these conditions are also common; anxiety disorders affect nearly 29% of individuals across the life course, while mood disorders (e.g., depression and bipolar disorder) affect nearly 21% (Kessler et al., 2005). They also tend to recur and intensify with additional stressors, contributing through overlapping mechanisms such as emotional dysregulation, psychological pain, hopelessness, and impulsivity (Nock et al., 2010). Because these concerns frequently co-occur, diagnosis alone may not fully explain repeated suicidal behavior, highlighting the importance of examining their independent contributions for prevention.

### Qualitative research and suicide attempts

Existing research on suicide risks has made substantial progress in identifying who is more likely to reattempt suicide, but it is less equipped to explain *why* suicidal crises occur, *when*, and *how* they do. Although structured suicide assessments such as the Beck Scale for Suicide Ideation (BSI; Beck et al., 1979) and the Columbia Suicide Severity Rating Scale (C-SSRS; Posner et al., 2008) assess key clinical indicators, their predictive accuracy has not substantially improved in decades (Franklin et al., 2017). This limitation suggests that while psychiatric diagnoses are essential for understanding risk, they are insufficient on their own to determine who will reattempt and under what circumstances. This is because suicidal behavior often emerges from dynamic interactions among mental health symptoms, relational strain, situational stressors, and subjective experiences of isolation and disconnection (Nock et al., 2010). Accordingly, examining interpersonal and situational factors alongside psychiatric risk may provide a more complete understanding of repeated suicidal behavior, highlighting the value of qualitative approaches that capture the contextual dynamics surrounding suicide attempts.

Qualitative research offers a complementary lens by capturing how suicidal crises unfold within interpersonal and situational contexts. Studies drawing on suicide notes, medical records, police reports, and interviews have revealed recurring interpersonal and situational triggers, including loneliness, relationship problems, family conflict, chronic illness, substance use, and financial instability (Fallahi-Khoshknab et al., 2023; Guest et al., 2021; Jack et al., 2018; Sanger & Veach, 2008). The patterns identified in qualitative accounts align closely with the IPTS, particularly the constructs of thwarted belongingness and perceived burdensomeness, suggesting that the theory’s core mechanisms are not only theoretical but are reflected in first-person descriptions of suicidal crises.

### Integrating risk and meaning with mixed methods

Despite substantial advancements in identifying risk factors and documenting lived experiences, we still cannot explain why suicide attempts recur in specific moments, or how mental health concerns and interpersonal dynamics converge to produce these crises. Quantitative models identify who is at risk but struggle to capture the situational conditions under which attempts occur, while qualitative accounts reveal these dynamics but lack generalizability. As a result, a gap remains in understanding how risk and context operate together in shaping initial and repeated suicidal behavior.

Recent mixed-methods research underscores the value of narrative data. Kheibari et al. (2023) analyzed suicide-related law enforcement calls and found that while depression was noted in over 30% of incidents, their qualitative analysis of officers’ notes identified three additional interpersonal themes not captured through their quantitative design. These themes included references to self-harm or self-injurious behavior as a means of gaining control (e.g., threatening to harm oneself to achieve a desired outcome), minimizing suicidal intent (e.g., stating “I’m not suicidal, I’m just upset”), and promising safety (e.g., agreeing not to reattempt). Importantly, these narrative descriptions may reflect a range of intent, including non-suicidal self-injury, ambiguous intent, or suicidal behavior, and such distinctions are not always clearly documented in police reports. These findings suggest that critical interpersonal dynamics are not just underrepresented in structural data but are systematically missed without narrative context.

Therefore, building on Kheibari et al. (2023), the current study examines how interpersonal dynamics and mental health concerns—as documented in police reports—shape police documented suicide attempts and contribute to repeated behavior. Drawing on four years of police calls for service, we combine thematic analysis of officer narratives with logistic regression models of administrative incident data to examine both the contextual dynamics surrounding suicide attempts and the individual-level factors associated with repeated attempts. We focus specifically on *documented suicide attempts*, not broader suicide-related calls (i.e., suicidal ideation or death), to examine a clearly defined population, and analyze narratives that include statements from people who have attempted suicide, loved ones, and/or responding officers, offering interpreted accounts of crises. Therefore, we explore the subjective explanations individuals—or their associates—offer for suicidal behavior, while also assessing the documented presence of specific mental health conditions.

We address three main research aims: (1) to explore how suicide attempts are described and contextualized in officer narrative reports; (2) to examine whether police-documented mental health concerns are associated with repeated suicide attempts; and (3) to assess whether the odds of repeated attempts differ across specific mental health concerns. Aim 1 is exploratory and qualitative in nature, examining the interpersonal and situational dynamics of suicidal crises as documented by officers. Aims 2 and 3, while quantitative, are similarly treated as exploratory, given that prior research is mixed on the expected direction and ordering of effect sizes across specific mental health concerns (Abascal-Peiró et al., 2023; de la Torre-Luque et al., 2023). Although we do not formally test the IPTS, we use it as an interpretive framework to situate our findings within broader theoretical constructs related to interpersonal distress and suicidal behavior. Through this mixed-methods design, we aim to generate insights that are both theoretically grounded and actionable for suicide prevention, particularly in the context of first responders and crisis intervention.

## Methods

### Data and sample

Data for this study come from one mid-sized Midwestern police department in the U.S. and consist of roughly 15,800 total mental health-related incident reports generated between January 2018 and December 2021. Reports were written by officers responding to mental health-related calls, including incidents classified as sickness, overdose, injury, and suicide attempts. Each report includes two main components: (1) an officer narrative describing and summarizing the circumstances of the incident, and (2) an administrative section capturing details of the incident and involved parties. Narrative level-of-detail varied across officers and incidents. The officer narratives are non-systematic and reflect officer discretion; therefore, a ‘no’ on these variables derived from the narrative reflects ‘non-documentation’ rather than a true absence. The administrative section is standardized and required for officers; therefore, a ‘no’ is likely a true ‘no’ response rather than ‘non-documentation.’

Because these narratives are generated by responding officers rather than through clinical assessments or firsthand accounts from individuals experiencing suicidal behavior, they should be interpreted as officer-documented accounts of suicidal crises. The narratives reflect what officers observed and chose to document, are informed by statements from involved individuals and witnesses, as well as the circumstances of the encounter. Accordingly, these data provide insight into how suicidal crises are documented within law enforcement settings while also capturing interpersonal and situational information that is often unavailable in structured administrative records. This perspective is both a strength and limitation of the data, as the narratives provide rich contextual information but may differ from how individuals themselves or mental health professionals would characterize the same events. This study was deemed non-human subjects research by the Institutional Review Board at The University of Nebraska Medical Center and did not require approval.

To be included in the study, incidents had to be documented as suicide attempts. As the dataset included a broad range of mental health-related calls, the first step was to identify which incidents constitute suicide attempts. The lead author worked with two officers from the police department’s Behavioral Unit to develop classification criteria. According to the Behavioral Unit, an incident was classified as a suicide attempt only when both (a) explicit suicidal intent (e.g., statements such as “I wanted to die”) and (b) a self-injurious act (e.g., ingesting pills or cutting) were present in the police narrative. Incidents involving suicidal statements without a self-injurious act were excluded, as were incidents involving self-injurious behavior without documented suicidal intent. As such, non-suicidal self-injury (NSSI) was outside the scope of this study. Using these criteria, the lead author manually reviewed all reports.^1^ This process identified 2,659 suicide attempts involving 2,240 unique individuals over the four-year study period. One case had missing data on sex and was excluded from analyses, resulting in a final analytic sample of 2,658 incidents (~ 17% of total incidents).

Although police data are rarely used to study suicidal behavior (for exception, see Kheibari et al., 2023), incident reports offer several advantages. First, police are first responders to suicide-related calls (Lersch, 2020), despite reporting limited preparation for managing such crises (Kheibari et al., 2023; Kuehl et al., 2023). Second, officers typically write reports during their shift (Malm et al., 2007), capturing timely observations of an incident (Shernock, 2005). Third, the officer narrative section captures observed or recalled motivations and stressors surrounding the attempt (Green et al., 2023), offering a window into real-world contexts (Goldsmith et al., 2002; Kirmayer, 2022). Finally, administrative datasets provide large samples, thereby enhancing generalizability (Langton et al., 2021; Sherman, 2013). Descriptive statistics for all quantitative variables are displayed in Table 1.

**Table 1:** Descriptive statistics (*N* = 2,658)

	Mean / %	SD		Min	Max
Repeated Suicide Attempts	15.76%			0	1
Mental Health Concerns					
Total	19.60%			0	1
Depression disorder	12.49%			0	1
Bipolar disorder	6.21%			0	1
Anxiety disorder	4.85%			0	1
Control Variables					
Age (truncated)	32.62	15.10		8	92
Sex					
Male	41.31%			0	1
Female	58.69%			0	1
Race/Ethnicity					
White	64.90%			0	1
Black	18.85%			0	1
Hispanic	8.01%			0	1
Other/Unknown	8.24%			0	1
Reporting Party	47.86%			0	1
Witness Present	26.41%			0	1
Attempt Interrupted	11.81%			0	1
Substance Use	24.91%			0	1
Time Period					
Before COVID-19	53.72%			0	1
During COVID-19	46.28%			0	1

*Abbreviations.* *SD* standard deviation, *min* minimum, *max* maximum

### Quantitative measures

#### Repeat suicide attempts

The outcome of interest is *repeat suicide attempts*. Consistent with the Diagnostic and Statistical Manual of Mental Disorders, 5th addition (DSM-5) criteria, suicide attempts are operationalized as self-initiated behavior with the expectation that the behavior would lead to death (American Psychiatric Association [APA], 2013). A binary variable is created that indicates whether an individual had multiple suicide attempts during the study period: individuals with one documented attempt are coded 0, and those with two or more attempts are coded 1. Just over 15% of individuals have more than one suicide attempt during the study period. The four-year window (2018–2022) is appropriate for examining repetition, as most reattempts occur within 6–12 months of an initial attempt (Suárez-Pinilla et al., 2020).

#### Police documented mental health concerns

*All mental health concerns* are defined as any mental health concern and are coded as present (= 1) if the police narrative explicitly referenced a diagnosed or suspected mental health condition (e.g., psychiatric symptoms, treatment history, medication use, recent hospitalization, or prior diagnoses: “[son’s name] is diagnosed with depression and anxiety”). Only clear indicators are coded; vague references (e.g., “feeling depressed”) are excluded. Overall, 521 individuals (19.6%) are coded as having police documented mental health concerns. Because this measure depends on officer documentation, individuals with mental health concerns may be undercounted. Still, in the absence of clinical data, this approach provides a reliable and conservative method for capturing mental health indicators (Sarkar & Seshadri, 2014). Appendix A lists all mental health concerns included in the narratives.

Because many mental health conditions occurred infrequently, analyses of specific diagnoses were limited to depressive disorders, bipolar disorders, and anxiety disorders, which were the only categories with sufficient prevalence for statistical analysis. These categories were not mutually exclusive, and individuals could be coded as having more than one documented mental health condition.

*Depressive disorders* are defined in accordance with the DSM-5 as “the presence of sad, empty, or irritable mood, accompanied by somatic and cognitive changes that significantly affect the individual’s capacity to function” (APA, 2013, p. 155). Depressive disorders are coded as present (= 1) if the narrative explicitly mentions a diagnosis or treatment course of action (e.g., “I took my pills [Escitalopram] because I’m depressed and want to die” or “I have bipolar and depression but can’t take my meds [Sertraline] because I’m pregnant”). Vague statements of mood without diagnostic clarity (e.g., “I feel depressed”) and cases with no mood-related indicators are coded as 0. Of 2,659 suicide attempt incidents, 332 (12.49%) are coded as involving a depressive disorder (63.72% of all mental health concerns).

*Bipolar disorder* captures a period of:

“abnormally and persistently elevated, expansive, or irritable mood and abnormally and persistently increased goal-directed activity of energy, lasting at least 1 week and present most of the day, nearly every day or any duration if hospitalization is necessary” (APA, 2013, p. 125).

Bipolar is coded as present (= 1) if the narrative references a diagnosis (e.g., “I’ve had bipolar for 10 years now”) or bipolar medications (e.g., “I took my Depakote [bipolar medication] to kill myself”). Cases without diagnostic specificity are coded as not present (= 0). A total of 165 suicide attempt incidents (6.21%) involve bipolar disorder (31.67% of all mental health concerns).

*Anxiety disorder* is defined as “excessive anxiety and worry (apprehensive expectations), occurring more days than not for at least 6 months, about a number of events or activities, such as work or school performance” (APA, 2013, p. 222) and is coded as present (= 1) if the narrative references a diagnosis (e.g., “I have severe anxiety disorder”) or anti-anxiety medications (e.g., Xanax, Ativan, Lexapro) in contexts confirming an underlying disorder. General or situational distress without diagnostic clarity is coded as absent (= 0). Overall, 129 suicide attempt incidents (4.85%) involve an anxiety disorder (24.76% of all mental health concerns).

#### Control variables

To reduce model misspecification, we include a range of demographic and situational controls. All demographic measures are drawn from the administrative section of incident reports. We control for *age* measured in years at the time of the suicide attempt (*mean* = 32.62, *SD* = 15.10, *min* = 8, *max* = 92) and *male* (= 1; 41.31%), where female is the reference category (= 0; 58.69%). *Race/ethnicity* is coded using mutually exclusive indicators for *Black* (18.85%), *Hispanic* (8.01%), and *Other/Unknown* (8.24%), with *White* (64.90%) as the reference.^2^

Situational control variables are drawn from officer narratives, which are non-systematic rather than standardized. All variables are binary and coded as ‘yes’ when it was explicitly documented in the narrative and ‘no’ when they were not documented. First, three variables that capture the protective potential of social presence are included: *reporting party present* (i.e., attempted or other individual calls 911 = 1; 47.86%); *witness present* (= 1; 26.41%); and *attempt interrupted* before completion (= 1; 11.81%). These elements may strengthen support, facilitate treatment, and reduce access to lethal means (Dazzi et al., 2014; Gould et al., 2022; Hu et al., 2023; Kleiman & Liu, 2013; O’Connell et al., 2024). Second, *substance use* is coded as present (= 1) if alcohol and/or drug use was observed, self-reported, or observed by a witness (24.91%). Finally, to account for COVID-19 pandemic-related effects (e.g., stress, isolation, mental health challenges; Biddle et al., 2013; Dubé et al., 2021; Koziarski, 2021), an indicator is included for whether the incident occurred before (= 0; 53.72%) or after (= 1; 46.28%) the onset of the pandemic (i.e., March 11, 2020; WHO, 2020), with incidents occurring on March 11, 2020 coded as post-pandemic.

### Analytic strategy

#### Qualitative analysis – aim 1

To enrich our understanding of the interpersonal and situational context surrounding suicide attempts, we first conduct a qualitative thematic analysis of the officer narrative sections of the incident reports. Reports are included if the officer’s narrative section contains a substantive description of the circumstances surrounding the suicide attempt (e.g., individual’s statements, situational context, behaviors). Officer narratives reflect what responding officers chose to document and include both verbatim and paraphrased statements. Reports consisting solely of administrative notes (e.g., dispatch logs) were excluded, leaving 1,592 reports (~ 60% of the total) for the qualitative analysis.

We use an inductive, iterative coding approach consistent with thematic analysis procedures (Braun & Clarke, 2006). Narratives from 2018 were first read to identify emerging patterns and develop preliminary codes. Through open coding and constant comparison (Glaser & Strauss, 1967), the data were reviewed repeatedly until no new codes emerged. These initial codes were then refined and organized into broader themes that capture recurring psychosocial influences on suicidal behavior. To ensure consistency, the 2018 narratives were re-coded using the finalized coding scheme, which was then systematically applied to narratives from subsequent years. All data coding was conducted in Excel by the lead author.

#### Quantitative analysis – aims 2 & 3

To address Aim 2, which examines whether police-documented mental health concerns are associated with reattempts, and Aim 3, which assesses if the odds of reattempts differ across mental health concerns, we estimate four logistic regression models predicting repeat suicide attempts—a binary variable (Cox, 1958). Our analytic strategy proceeds in two stages. First, we estimate a logistic regression model predicting repeat suicide attempts using all mental health concerns. Second, we run three separate models for depressive disorder, bipolar disorder, and anxiety disorder. To explore whether associations differ across these models, we conduct equality-of-coefficients tests for dependent groups to compare coefficients (Paternoster et al., 1998). These comparisons are interpreted descriptively because logistic regression coefficients are scale-dependent.

All models include demographic and situational control variables to adjust for confounding differences across individuals.^3^ Robust standard errors are clustered at the individual level to account for 419 individuals who appear in the dataset more than once (Allison, 1999). While clustering adjusts the standard errors to account for non-independence, it does not model within-person change over time or unobserved individual differences that may influence repeated attempts. The analytic sample includes 2,658 incidents; no missing data were present. Analyses were conducted in Stata 18 (StataCorp, 2023).

## Results

### Qualitative results: thematic analysis

The frequencies reported below reflect what officers at the scene observed and documented; therefore, individuals may have experienced additional stressors or interpersonal dynamics that were present but not captured in the narrative report. As in prior research demonstrating that narrative data capture interpersonal and contextual dynamics not reflected in structured measures (e.g., Kheibari et al., 2023), these findings highlight dimensions of suicidal behavior that extend beyond administrative data. Through thematic analysis, we identified seven non-mutually exclusive themes that illuminate individuals’ motivations for non-fatal suicide attempts and the contexts in which these crises occur. We also examine how these themes appear among individuals with police documented mental health concerns. Of the 521 individuals with such documentation, 376 had sufficient details for the qualitative analyses (see Appendix B for more detail).

### Romantic relationship problems

The most frequently cited motivation for suicide attempts involved romantic relationships (27% of the narrative reports; *n* = 428/1,592). This theme was the second most prevalent among individuals with documented mental health concerns (24%; 90/376). Individuals often described recent breakups or divorce as emotionally destabilizing. One individual, who wanted to jump off a bridge, explained that her boyfriend had just broken up with her, and she “just want[ed] to end it.” Another individual had recently gone through a breakup and stated: “I just want my boyfriend to like me.” Some framed these events as abandonment or rejection, such as a wife responding to a husband’s suggestion of separation with, “I won’t live without you, and guess what, I took those nitro [Nitroglycerin] pills.” Another individual was having marital issues and fighting with his wife and said, “I shot myself in the mouth. I’m in the bathroom. I shot myself.” These narratives suggest that romantic relationship disruptions were not experienced as isolated interpersonal events, but as acute threats to belonging and attachment security.

### Mental and physical health concerns

Mental and physical health concerns were the second most documented reason for suicide attempts (20%; 324/1,592), and the most prevalent among individuals with documented mental health concerns (30%; 114/376), suggesting a strong association between clinical symptoms and suicide risk. Narratives referenced depression and substance use disorders. For example, one individual explained, “I have been depressed for the last year. Sometimes I think it’s better to just die.” Another individual sent a message to a friend on Facebook, saying, “I took a bunch of pills to kill myself. I’m not right and I know that. I want treatment for drug addiction or I’ll die.”

Other narratives described psychotic experiences, such as auditory hallucinations instructing individuals to self-harm. For example, one individual said, “I don’t want to kill myself but I know taking the pills would stop the voices, I took the pills to kill the voices.” Some accounts referenced chronic medical conditions (e.g., Crohn’s disease). One individual described “amplified pain disorder” and left a suicide note stating, “I felt like I wasn’t able to change my life enough to enjoy it again. I’m sorry. I didn’t want to leave everyone. I’m so lonely.” Importantly, these accounts suggest that psychiatric symptoms were often embedded within broader crisis contexts, rather than operating as isolated clinical risk factors.

### Family conflict

Ongoing or acute family conflict (e.g., arguments, role disputes, and caregiving responsibilities) was the third most prevalent theme among the full sample (18%; 282/1,592) and individuals with documented mental health concerns (14%; 54/376). Both youth and adults cited disputes with parents, children, or other relatives as key stressors. For example, a woman said after arguing with her daughter, “This is why people commit suicide,” before walking away and attempting suicide. Another individual had an argument with their uncle (as documented in the police report) and left their mother a message saying:

When y’all find my dead body one day don’t say I didn’t warn y’all. Shit, at least you will have $50,000 to buy yourself something nice. Get you a car. Know that you have always been the best mother and it’s not your fault. I just want to go.

### Loneliness and worthlessness

Feelings of isolation, rejection, and worthlessness often appeared in the narrative reports (14%; 219/1,592), and among individuals with documented mental health concerns (13%; 48/376). Individuals expressed feelings of being unloved, unwanted, or entirely alone in the world. Examples include, “I feel worthless and depressed,” “I feel worthless and it’s been this way for a long time. I just want to end my life,” and “I want to join my brother [in heaven] because no one cares about me.” Some directly stated that they felt like a burden on their families, linking interpersonal disconnection with diminished self-worth: “I am depressed because I feel like a burden to my father [who they live with].” These themes resonate acutely with the IPTS.

### General life struggles

Participants also described cumulative general life stressors such as financial hardship, unstable housing, school and work pressures, and unresolved grief (13%; 209/1,592), which was also present among individuals with documented mental health concerns (12%; 44/376). For example, one individual reported being overwhelmed by a combination of the death of her boyfriend, overdue rent, and not making enough money; she left messages to her children saying, “I love all of you guys but the pain is too much for me anymore, love mommy.” She also sent a message to her boss referring to living by saying, “It’s not worth it.” Another individual, facing similar financial struggles, left a message saying:

It will all be over tonight. I can’t do this anymore. I can’t lose another job. The plan is in motion, I’m sitting in my garage [with the car running to asphyxiate], I can’t face work tomorrow, and if I leave my garage, I would take all my pills.

While not always acutely triggering, these challenges appeared to build over time, leading some individuals to view suicide as a means of escape. One subject, who experienced a breakup, was living in a friend’s basement, and did not receive a job he was a top candidate for, sent a message to his recent ex-girlfriend saying: “I just want to see you one last time, and you were wrong. I can’t forgive myself for what I did. I’m sorry. I will see you in the next life.” He also left a suicide note that read: “I want to escape the prison of my mind, the prison of who I became. You will be much better off without me.”

### Loss and bereavement

Experiences of loss, whether the death of a family member, friend, or pet, were destabilizing for some individuals in the full sample (8%; 121/1,592) and those with documented mental health concerns (6%; 24/376). One person, whose grandmother recently passed away, stated: “I need help. I just want to end it. I miss her [grandmother] so much, I want to be with her.” Others described bereavement following suicide deaths, pregnancy loss, or multiple losses. For example, individuals stated, “Just kill me, I want to die. My dad just committed suicide, and I can’t take this anymore” and “I lost my baby, and I don’t want to live anymore.” Another individual, who experienced both the loss of their father and friend within a few months, left a note saying:

I’m so sorry, please don’t blame yourself, you tried so hard to help me. It was all just too much for me and I don’t feel like going on anymore. You can hate me, it’s okay. I just don’t want to go on anymore.

### COVID-19 pandemic stress

Although less common, a small number of people in the full sample (1%; 9/1,592), and individuals with documented mental health concerns (1%; 2/376), explicitly attributed their suicide attempts to stressors related to the COVID-19 pandemic, including fear of illness, job loss, online schooling, social isolation, and uncertainty about the future. For example, one individual expressed concern about their grandmother being diagnosed with COVID-19 and said, “I’m worried about my grandma. I don’t want to live anymore.” Rather than suggesting that concern for a loved one directly precipitated suicidal behavior, this example illustrates how the uncertainty, isolation, and emotional distress surrounding the pandemic may have intensified existing feelings of hopelessness and despair. Consistent with the IPTS, these stressors may have contributed to perceptions of burdensomeness, disconnection, or psychological pain that increased suicide risk.

Another example is a mother who had no indication of mental health concerns, stated: “I want to kill myself and my [daughter’s name] would be better off without me.” The daughter could not visit because they were quarantined due to the COVID-19 pandemic.

### Integrating themes with IPTS

The IPTS (Joiner, 2005) provides a useful interpretive lens for organizing the themes identified above. The narratives reveal consistent echoes of thwarted belongingness through expressions of loneliness, rejection, and loss. Perceived burdensomeness is reflected in statements such as “better off without me” and often co-occurs with financial struggles and caregiver situations. These experiences did not occur in isolation and were embedded in situational stressors such as chronic health concerns and cumulative life difficulties.

Across incidents, these themes were not mutually exclusive. Several incidents reflect multiple simultaneous stressors, such as mental health concerns alongside bereavement. For example, one person described experiencing psychotic symptoms, stating, “I’ve seen the devil and have heard his voice,” and also referenced a loss, stating, “I want to go to my mom,” who officers documented as deceased, “she’s in heaven.” This overlap suggests that suicidal crises documented in police narratives are best understood as multidimensional events involving intersecting interpersonal, psychological, and situational stressors rather than isolated triggers.

The repeated exposure to distress, trauma, and self-harm demonstrates the ongoing mental health struggles and prior attempts that may be associated with developing an acquired capability of suicide. Relationship conflict and acute interpersonal crises act as triggers to underlying vulnerabilities. Taken together, these findings suggest that suicidal behavior is shaped by both individual and situational factors that align with the core concepts of the IPTS. Importantly, the findings highlight how these processes unfold in real time, offering insight into the immediate context of a suicide crisis.

### Quantitative results

Building on the qualitative findings, which focused on interpersonal and situational factors that shape suicide attempts, the quantitative analyses examine whether mental health concerns are associated with *repeated* attempts. Mental health concerns are a major risk factor for suicidal behavior (Nock et al., 2010), and the qualitative findings highlight that they are frequently mentioned in officers’ narratives. Yet their documentation in narratives and prevalence in the literature do not tell us whether mental health concerns predict reattempts specifically. The quantitative analyses directly address this gap by examining whether police-documented mental health concerns, and which specific concerns, are associated with higher odds of repeated attempts.

### Overall mental health and repeated suicide attempts

Table 2 presents the results from the logistic regression examining the association between mental health concerns and the odds of repeated suicide attempts, controlling for relevant covariates. The coefficient for overall mental health concerns is negative (*b*=-0.18, OR = 0.83), indicating that individuals with documented mental health concerns had approximately 17% lower odds of repeated suicide attempts compared to those without documented concerns. However, the association is not statistically significant (*p* = 0.24). The null findings should be interpreted with caution, as a response of ‘no’ for mental health concerns reflects non-documentation rather than a confirmed absence.

**Table 2:** Logistic regression predicting repeated suicide attempts by mental health (*N* = 2,658)

	b	(SE)	OR
Mental Health Concern	-0.18	0.15	0.83
Age	0.01	0.01	1.01
Male	0.06	0.18	1.06
Race *(ref: White)*			
Black	-0.41	0.27	0.67
Hispanic	-0.80*	0.33	0.45
Other/Unknown	-0.25	0.22	0.78
Reporting Party	-0.39**	0.12	0.68
Witness Present	-0.40*	0.18	0.67
Attempt Interrupted	0.22	0.23	1.25
Substance Use	-0.16	0.15	0.85
During COVID-19	0.25	0.13	1.29
Constant	-1.63	0.25	0.20

Logistic regression was estimated with a maximum likelihood estimator and robust standard errors

*Abbreviations. b* unstandardized coefficient, *SE* cluster robust standard error, *OR* odds ratio

*p < 0.05, **p < 0.01

### Depression, bipolar, anxiety disorders, and repeated suicide attempts

Table 3 presents the results from three logistic regression models assessing whether specific mental health concerns—depression disorder, bipolar disorder, and anxiety disorder—are associated with repeated suicide attempts, controlling for covariates. For depressive disorder, individuals with a documented condition have approximately 17% lower odds of repeated suicide attempts compared to those without (*b*=-0.19, *p* = 0.31, OR = 0.83). However, this association is not statistically significant. Similarly, individuals with bipolar disorder have about 8% lower odds of reattempts (*b*=-0.08, *p* = 0.71, OR = 0.92). Those with anxiety disorder have approximately 15% lower odds (*b*=-0.15, *p* = 0.57, OR = 0.85), neither of which reach statistical significance.

**Table 3:** Logistic regression predicting repeated suicide attempts by type of mental health (*N* = 2,658)

	Model 1			Model 2				Model 3		
	b	(SE)	OR	b	(SE)		OR	b	(SE)	OR
Depression	-0.19	0.18	0.83	—		—	—	—	—	—
Bipolar	—	—	—	-0.08		0.23	0.92	—	—	—
Anxiety	—	—	—	—		—	—	-0.15	0.27	0.85
Age	0.01	0.01	1.01	0.01		0.01	1.01	0.01	0.01	1.01
Male	0.06	0.18	1.06	0.06		0.18	1.07	0.06	0.18	1.06
Race (*ref: White*)										
Black	-0.40	0.27	0.67	-0.39		0.27	0.67	-0.40	0.27	0.67
Hispanic	-0.80*	0.32	0.45	-0.79*		0.32	0.45	-0.80*	0.32	0.45
Other/Unknown	-0.25	0.22	0.78	-0.25		0.22	0.78	-0.25	0.22	0.78
Reporting Party	0.39**	0.11	0.67	-0.40**		0.11	0.67	-0.39**	0.11	0.67
Witness	-0.40*	0.18	0.67	-0.40*		0.18	0.67	-0.40*	0.18	0.67
Attempt Interrupted	0.22	0.23	1.24	0.20		0.23	1.24	0.22	0.23	1.24
Substance Use	-0.16	0.15	0.85	-0.17		0.15	0.85	-0.16	0.15	0.85
During COVID-19	0.25	0.13	1.28	0.25		0.13	1.28	0.25	0.13	1.28
Constant	-1.64	0.26	0.19	-1.67		0.26	0.19	-1.66	0.26	0.19

Logistic regression was estimated with a maximum likelihood estimator and robust standard errors

*Abbreviations. b* unstandardized coefficient, *SE* cluster robust standard error, *OR* odds ratio

*p < 0.05, **p < 0.01

To assess whether these effects differ across diagnoses, we conduct equality-of-coefficients tests comparing the three models (Paternoster et al., 1998).^4^ Results indicate no significant differences across diagnoses. The effect of depression does not differ from bipolar disorder (χ²=0.19, *p*=0.66), depression does not differ from anxiety (χ²=0.02, *p*=0.90), and bipolar disorder does not differ from anxiety (χ²=0.05, *p*=0.82). Although none of the models reached statistical significance, the odds ratios are similar in magnitude (i.e., consistently below 1.00), even though the three diagnoses are statistically indistinguishable in their association with repeated attempts.

## Discussion

This study provided a closer look at how suicide attempts that resulted in police response unfold in real-world crisis contexts, particularly among individuals who go on to reattempt. Drawing on law enforcement narratives alongside administrative data, the findings move beyond identifying who is at risk to examining the interpersonal and contextual conditions present at the time of the attempt. Given that individuals who survive an initial suicide attempt face a substantially elevated risk of subsequent attempts compared to individuals without a prior suicide attempt (de la Torre-Luque et al., 2023; Ribeiro et al., 2016), distinguishing what characterizes these events is critical. The results suggest that suicidal crises are shaped not only by documented mental health concerns, but also by immediate relational and situational dynamics captured in officer narratives. Several key findings emerged from this analysis.

The thematic analysis revealed that suicidal crises documented by police rarely reflect a single, isolated trigger. Instead, officer narratives describe overlapping stressors such as relationship conflict, financial strain, health struggles, and emotional distress, suggesting that attempts emerge from the accumulation of multiple simultaneous pressures rather than any one factor alone. This pattern aligns with key components of the IPTS (Joiner, 2005; Van Orden et al., 2010), particularly expressions of thwarted belongingness (e.g., “I’m so lonely”) and perceived burdensomeness (e.g., “I feel like a burden to my father”). Notably, individuals with police-documented mental health concerns often attributed their suicidality to internal distress. In contrast, narratives without documented mental health concerns focused more on interpersonal or situational stressors. This pattern indicates that mental health is not merely a background risk factor but an actively experienced component of the crisis itself. This suggests that mental health may shape how individuals interpret and respond to interpersonal and situational stressors, amplifying experiences of relationship conflict, isolation, or financial strain into perceived hopelessness or burdensomeness in ways that elevate crisis risk.

Contrary to expectations, mental health concerns were not associated with repeated suicide attempts. However, this finding may reflect measurement limitations, small sample size, and a broader pattern observed in the qualitative data. Officer documentation of mental health varies widely due to differences in training, time constraints, stigma, and individual discretion (Human Rights Watch [HRW], 2019; Kheibari et al., 2023; Moradinazar et al., 2017), meaning the absence of documentation may not necessarily indicate the absence of mental health concerns. At the same time, the qualitative findings suggest that suicidal crises are rarely driven by a single identifiable factor. If attempts are shaped by overlapping interpersonal and contextual stressors, isolating mental health documentation alone may be insufficient to distinguish individuals who reattempt.

Similarly, the odds of repeated attempts did not differ across types of documented mental health concerns (i.e., depression, bipolar, and anxiety). While prior meta-analyses have consistently found associations between each of these conditions and reattempts, the relative strength of those associations has been inconsistent across studies (Abascal-Peiró et al., 2023; de la Torre-Luque et al., 2023). The absence of differences across conditions in the present study could be due to different measurement context, officer documentation compared to clinically assessed diagnoses using standardized instruments, rather than a true absence of association. At the same time, this finding is consistent with broader evidence that psychiatric diagnoses alone have limited predictive utility for suicidal outcomes (Carter et al., 2017; Franklin et al., 2017).

Taken together, the qualitative and quantitative findings are complementary in demonstrating that structured police data provide an incomplete representation of suicidal crises. Administrative fields captured basic indicators, while officer narratives revealed interpersonal and contextual dynamics that appear central to how these crises unfold. Importantly, as these findings are derived from officer-generated narratives, they should be interpreted as reflecting suicidal crises as they are documented during law enforcement encounters rather than as direct representations of individuals’ lived experiences. Police involvement typically occurs during acute crises requiring immediate safety intervention, often following reports from family members, bystanders, or other community members, which may differ from attempts managed through healthcare settings or those that never come to official attention. Officers are responsible for rapidly assessing safety, stabilizing the immediate situation, and determining an appropriate disposition rather than conducting comprehensive clinical evaluations. At the same time, these narratives provide a unique perspective on how suicidal crises come to the attention of law enforcement and the contextual information officers document during those encounters. The absence of this contextual information from standardized reporting represents a missed opportunity for both research and intervention. Improving how these dynamics are documented, either through standardized reporting practices or the integration of narrative and clinical data, may enhance both the understanding and prevention of suicide attempts (Kheibari et al., 2023).

These findings also have policy and practice implications. Because police are often first responders to behavioral health crises, understanding how these encounters are documented has important implications. Prior research has noted that officers often feel undertrained and underprepared for mental health crisis calls (Chidgey et al., 2019; Osteen et al., 2020), and this study’s findings reinforce the need for training that emphasizes not only behavioral indicators but also interpersonal and situational warning signs. Understanding both what officers observe and what may remain undocumented has important implications for improving crisis response, documentation practices, and continuity of care. Crisis Intervention Team (CIT) and co-responder models, which integrate mental health professionals into police response systems, are widely implemented approaches to crisis response; however, evidence regarding their effectiveness is mixed (Marcus & Stergiopoulos, 2022; Oblath et al., 2025). Recent research highlights the growing development of civilian-led crisis response alternatives that operate outside traditional law enforcement structures and represent a potential shift in how behavioral health emergencies are addressed (Giacomantonio & Horn, 2026). More broadly, expanding access to community-level mental health resources, such as the 988 Suicide and Crisis Lifeline, may help reduce reliance on police intervention by providing support before a crisis escalates. For individuals who have already attempted suicide, structured hospital follow-up protocols and evidence-based treatments, including suicide safety planning (Rogers et al., 2022), cognitive behavioral therapy (Rudd et al., 2015), and coordinated aftercare (Exbrayat et al., 2017) remain critical tools for reducing the risk of reattempt, particularly given the elevated risk faced by those with a history of prior attempts.

Several limitations should be noted. First, reliance on police narratives introduces variability due to differences in officer training, discretion, and reporting practices (Kheibari et al., 2023; Schade & Thielgen, 2022), potentially affecting how mental health concerns and situational factors are captured. Second, these data are limited to suicide attempts that came to police attention and therefore exclude attempts that did not involve police contact. As a result, suicide reattempts may be undercounted, and the findings may not generalize to suicide attempts that do not involve a law enforcement response (Moradinazar et al., 2017). Third, because the dataset is limited to a defined observation window and jurisdiction, some individuals classified as single attempters may have had prior or subsequent attempts that were not captured in these records. Linking police records to hospital or emergency department data would improve classification accuracy and provide a more complete account of attempt history. Finally, the cross-sectional research design limits inferences about temporal ordering between mental health concerns and suicide attempts. It is also possible that mental health documentation reflects repeated system contact rather than underlying psychiatric status as officers may be more likely to document mental health concerns for individuals with multiple encounters or more serious incidents. Future longitudinal research linking police records to clinical data would address these limitations.

## Conclusion

Suicide research has often focused on *who* is at risk, whereas this study’s contribution adds to the understanding of *why* people become suicidal and *how* suicide crises are captured by law enforcement, a critical gap to address. Police narratives, despite their limitations, capture interpersonal and situational elements of these moments that structured data routinely miss. The qualitative findings reveal that some officers do capture rich interpersonal and situational accounts of suicide attempts, helping shed light on why some people become suicidal, which often results from overlapping stressors. The null quantitative findings suggest that when mental health is measured solely through officer documentation from a single encounter, its predictive utility may be limited, potentially because this measure does not fully capture the complexity of mental health. Together, the results demonstrate the importance of mixed-methods approaches for police data research, particularly for understanding what information law enforcement captures and misses regarding suicidal crises. This means systematically capturing situational context and providing mental health care to individuals experiencing multiple crises and stressors. The more we understand not only who is at risk but *why* people attempt suicide, the greater our potential to develop interventions that save lives.

## Data Availability

The data supporting the findings of this study are not publicly available and cannot be shared because they contain highly sensitive information that could compromise participant privacy and confidentiality. Data access is also restricted by the terms of a data-sharing agreement with the participating police department.

## Appendices

### Appendix A Mental health concerns from police narratives

**Table:**

Mental Health Concern	Frequency (f)	Percentage Among the Full Sample (%)
Depression	332	12.49
Bipolar	165	6.21
Generalized Anxiety	129	4.85
Post-Traumatic Stress Disorder	71	2.67
Schizophrenia	52	1.96
Attention Deficit Hyperactivity Disorder	23	0.87
Attention Deficit Disorder	12	0.45
Borderline Personality Disorder	12	0.45
Autism	11	0.41
Schizoaffective Disorder	9	0.34
Substance Use Disorder	8	0.30
Intellectual Disability	6	0.23
Oppositional Defiant Disorder	6	0.23
Major Depression	5	0.19
Panic disorder	5	0.19
Postpartum Depression	4	0.15
Mood Disorder	4	0.15
Psychosis	4	0.15
Severe Depression	3	0.11
Dementia	3	0.11
Obsessive Compulsive Disorder	2	0.08
Asperger Syndrome	1	0.04
Disruptive Mood Dysregulation Disorder	1	0.04
Agoraphobia	1	0.04
Separation Anxiety	1	0.04
Social Anxiety	1	0.04
Split Personality Disorder	1	0.04
Antisocial Personality Disorder	1	0.04
Multiple Personality Disorder	1	0.04
Emotional Diagnosis	1	0.04
Attachment Disorder	1	0.04

### Appendix B Qualitative themes among narratives

**Table:**

	Full sample(*N* = 1,592)	Mental health subsample(*n* = 376)	Non-mental health subsample(*n* = 1,216)
Theme 1: Relationship problems	27%	24%	28%
Theme 2: Mental and physical health concerns	20%	30%	17%
Theme 3: Family problems	18%	14%	19%
Theme 4: Loneliness and worthlessness	14%	13%	14%
Theme 5: General life struggles	13%	12%	13%
Theme 6: Loss or death	8%	6%	8%
Theme 7: COVID-19	1%	1%	1%

## Footnotes

1 If the first author was unsure whether a report should be classified as a suicide attempt based on the classification criteria, the incident was reviewed collaboratively with officers. After discussion, a majority vote was used to determine if an incident should be classified as an attempt.

2 2.86% of individuals had a race/ethnicity categorization of unknown. An additional 0.53% of individuals were categorized as other.

3 Prior to estimating results, we evaluate key model assumptions (Stoltzfus, 2011). Multicollinearity is assessed using Variance Inflation Factors (VIFs), all of which fell below the recommended threshold of 5 (range = 1.0 to 1.2), which indicates no concerns regarding collinearity among predictors (O’Brien, 2007). Linearity between continuous variables and the logit is examined using the Box–Tidwell procedure (Box & Tidwell, 1962), with no violations detected because the interaction terms were non-significant. Model fit is evaluated using the Hosmer–Lemeshow goodness-of-fit test (Hosmer et al., 2013); non-significant results across models indicate acceptable fit. Influential cases are assessed using Cook’s distance and leverage diagnostics, and no influential outliers were identified.

4 We used the ‘suest’ command in Stata to conduct the equality of coefficients test by comparing coefficients across the three models. Because our models for depressive disorder, bipolar disorder, and anxiety disorder are based on the same sample of individuals, the estimates are not independent. The suest command accounts for the shared variance to conduct the equality of coefficients test (Paternoster et al., 1998).
